# Sleep as a Priority: 24-Hour Movement Guidelines and Mental Health of Chinese College Students during the COVID-19 Pandemic

**DOI:** 10.3390/healthcare9091166

**Published:** 2021-09-06

**Authors:** Kaixin Liang, Clarice Maria de Lucena Martins, Si-Tong Chen, Cain Craig Truman Clark, Michael Joseph Duncan, He Bu, Liuyue Huang, Xinli Chi

**Affiliations:** 1 School of Psychology, Shenzhen University, Shenzhen 518060, China; liangkaixin2020@email.szu.edu.cn (K.L.); liuyue_huang@163.com (L.H.); 2 Research Centre in Physical Activity, Health, and Leisure—CIAFEL, Laboratory for Integrative and Translational Research in Population Health (ITR), Porto University, 4500 Porto, Portugal; claricemartinsufpb@gmail.com; 3 Department of Physical Education, Federal University of Paraiba, João Pessoa 58000-000, Brazil; 4 Institute for Health and Sport, Victoria University, Melbourne 8001, Australia; sitong.chen@live.vu.edu.au; 5 Faculty of Health and Life Sciences, Coventry University, Coventry CV1 5FB, UK; ad0183@coventry.ac.uk (C.C.T.C.); aa8396@coventry.ac.uk (M.J.D.); 6 Department of Social and Behavioural Sciences, City University of Hong Kong, Kowloon, Hong Kong, China; hebu22-c@my.cityu.edu.hk

**Keywords:** lifestyle, physical activity, sedentary behavior, sleep, mental health, COVID-19

## Abstract

Research on the combined role of 24-hour movement behaviors (sleep, sedentary behavior [SB], and physical activity) in adult mental health, though important, is in its infancy. In the context of Canadian 24-hour movement guidelines integrating quantitative recommendations for sleep, SB, and moderate-to-vigorous physical activity (MVPA), this study aimed to examine the associations between meeting guidelines and mental health among college students. The study used a cross-sectional sample of 1846 Chinese college students surveyed online in August 2020. Through network analysis and multivariate analysis of covariance, the individual and combined associations between meeting 24-hour movement guidelines and the levels of depression and anxiety after adjusting sociodemographic factors were analyzed. Results indicated that meeting the sleep guideline had stronger associations with depression and anxiety than meeting the SB or MVPA guideline. Specifically, compared to meeting no guidelines, meeting the sleep guideline (alone or in combination with other guidelines) was associated with significantly lower levels of depression and anxiety; meeting both SB and MVPA guidelines was also associated with a significantly lower level of depression. Hence, meeting more guidelines, especially adhering to a healthy sleep routine, may play an important role in promoting the mental health of young adults.

## 1. Introduction

Since the outbreak of the novel coronavirus (COVID-19), the pandemic has caused extraordinary life changes and stress. Concomitant to the unprecedented changes (e.g., quarantine measures and fear of being infected), aggravated public mental health problems have been reported in all age groups [1,2], especially college students [3,4]. Studies have reported that after the COVID-19 outbreak, the prevalence of depression and anxiety symptoms among Chinese college students was up to 56.8% and 41.1%, respectively [5,6]. Such a phenomenon may be related to home confinement widely adopted during the pandemic [7]. Considering that COVID-19 remains an unparalleled and ongoing crisis, it is essential to clarify the critical modifiable factors of mental health problems among college students to implement interventions appropriate to relieve and manage their mental burden. 

Sleep, sedentary behavior (SB), and physical activity (PA) have been identified as independent factors of health in college students. Daily hours of individuals are distributed among sleep, SB, and PA (also referred to as 24-hour movement behaviors). Hence, their individual or combined effects on health outcomes have received increasing attention from researchers. A large body of literature has illustrated the individual role of each 24-hour movement behavior in mental health among college students, even during the COVID-19 pandemic [3,8]. For example, college students with short sleep durations reported more depression symptoms than their counterparts [8]. A large-scale longitudinal study reported that less PA was a significant contributor to develop negative psychological symptoms among college students [3]. Relative to PA, SB was less studied but also considered to be an important factor affecting mental health during the pandemic [9]. Of note, in the field of time-use epidemiology, researchers have suggested that 24-hour movement behaviors are codependent given that the increase of one behavior would lead to the decrease of another behavior [10]. More importantly, time allocation may have important impacts on a series of health outcomes [10]. Hence, researchers have begun to adopt an integrated perspective to study the health implications of 24-hour movement behaviors across populations of all ages (e.g., children and adolescents) in recent years. Guided by this integrated movement paradigm and evidence from this field, Canada has first developed and released 24-hour movement guidelines for specific age groups, including quantitative recommendations for sleep, SB, and moderate-to-vigorous physical activity (MVPA) [11,12,13]. Canadian 24-hour movement guidelines acknowledge that focusing on a single behavior has limitations and suggest that a combination of sleep, SB, and PA matters for healthy development. Since the release of the guidelines, researchers have carried out studies to explore the association between guidelines adherence and some health-related outcomes (e.g., adiposity, fitness, quality of life) [14,15].

Because this is a relatively new topic, there are some research gaps to be addressed. First of all, compared to the abundant research on the association between meeting guidelines and better physical health [16], little is known about whether meeting guidelines is also related to better mental health. In addition, owing to the relatively late publication of the adult version of Canadian 24-hour movement guidelines, existing literature on this topic is mainly focused on children and adolescents [15] and findings from adults are relatively limited. Furthermore, less is clear about the relative importance of each recommendation in the guidelines, which would be particularly critical to design effective interventions. A recent study of Slovenian adults showed that associations between 24-hour movement guidelines and stress were mainly explained by meeting the sleep guideline, rather than the MVPA or SB guideline [17]. Nonetheless, more research is needed to establish the specific association between meeting guidelines and mental health among adults. Additionally, the guidelines are based on the research results before the COVID-19 pandemic [12], so it has yet to verify the desirable role of meeting guidelines in health during the pandemic. Moreover, the relationships between specific movement behavior and health outcomes may be affected by other movement behaviors [18,19] and sociodemographic factors [15,20]. To take these confounding variables into account synchronously, network analysis may be an applicable approach to reveal the relations between movement behaviors and health outcomes in an intuitive way. Network analysis aims to establish connections through multiple interactions between variables from graphical representations [21]. From a network perspective, we can better understand connections between variables in a complex system. Indeed, to facilitate a better understanding of the collective impact of 24-hour movement behaviors on health, researchers have begun to introduce network analysis into this field [22]. 

This study, therefore, aimed to investigate the associations between 24-hour movement behaviors (in isolation or combination) and mental health problems in Chinese college students during the COVID-19 pandemic. 

## 2. Methods

### 2.1. Participants and Procedure

The current cross-sectional study was conducted with an online survey in August (21st–31st) 2020. Due to the pandemic, Chinese college students spent the spring semester of 2020 in the form of online classes and confronted the uncertainty of academic and career development until September 2020, when they could return to educational settings for the fall semester. Therefore, at the time of our survey, most Chinese college students had stayed at home for more than six months. We adopted a convenient sampling method to recruit Chinese college students as participants via social media platforms (e.g., Wechat, QQ). Participants were asked to complete a questionnaire via “Wenjuanxing” (https://www.wjx.cn/, accessed on 3 September 2021), a Chinese online survey platform. Participants provided online consent before filling out the questionnaire. Those who had completed the questionnaire (approximately 15 min to finish) were given ten RMB (Chinese currency) via online payment as a gratuity for their time taken to respond. In total, 1942 students, from 30 provinces and autonomous regions (mainly from the Guangdong province), participated in the survey, and 1846 (response rate = 95.1%) provided valid answers and composed the final analytical sample. 

### 2.2. Measures

#### 2.2.1. Sociodemographic Factors

Participants reported sociodemographic information, including age (years), gender (male/female), body mass index (BMI), family structure (full/divorced/other), parental educational level (middle school or below/high school/college or university/master or above), number of siblings (none/one or more), number of friends (none/one to two/ three to five/six or more), residence (urban/rural), and perceived family affluence. Perceived family affluence was assessed by the MacArthur Scale [23,24]; the total score of the scale ranges from 1 (bottom rung) to 10 (top rung), with higher scores indicating higher perceived family affluence.

#### 2.2.2. 24-Hour Movement Behaviors

PA and SB were assessed via the Chinese version of the International Physical Activity Questionnaire—Short Form (IPAQ-SF) [25]. The IPAQ-SF asks participants to recall aspects of their PA over the past seven days, including the time spent on sitting (SB), walking, moderate PA, and vigorous PA. In the current study, MVPA time was represented by the total weekly accumulation of minutes spent on vigorous and moderate PA. Sleep duration was measured by the question from the Chinese version of the Pittsburgh Sleep Quality Index (PSQI): “During the past month, how many hours of actual sleep did you get at night?” [26]. Chinese versions of IPAQ-SF and PSQI have been widely used among the Chinese population and have shown good psychometric properties [27,28,29,30]. According to the Canadian 24-Hour Movement Guidelines for Adults aged 18–64 years, college students should achieve sufficient sleep (7 to 9 h per night), minimize SB (accumulated 8 h or less per day), and be physically active (at least 150 min of moderate to vigorous PA [MVPA] per week) in a healthy 24 h, so participants who reach criteria above were considered as meeting the 24-hour movement guidelines.

#### 2.2.3. Mental Health Problems

The level of depression was measured by the Chinese version of the 9-item Patient Health Questionnaire (PHQ-9) [31]. Each item was reported with a 4-point Likert scale, and a higher total score indicated a more severe level of depression symptoms. Total scores of 5, 10, 15, and 20 were the cut-off scores of mild, moderate, moderately severe, and severe levels of depression. The level of anxiety was evaluated by the Zung Self-rating Anxiety Scale (SAS) in the Chinese version [32]. The SAS consists of 20 items, each rated by a 4-point Likert scale. After multiplying the raw score by 1.25, the integer part is retained to obtain the standard score, and a higher standard score suggests a more severe level of anxiety. Total scores of 50, 60, and 70 were the cut-off scores of mild, moderate, and severe levels of anxiety. The Chinese versions of the PHQ-9 and SAS are both reliable instruments and have been widely used among Chinese adults [30,33,34,35]. The Cronbach alphas for the PHQ-9 and SAS in this study were 0.91 and 0.87, respectively.

### 2.3. Data Analyses

#### 2.3.1. Network Analysis

We specified two networks to model relations between 24-hour movement guideline adherence and mental problems separately for depression and anxiety. Each network also included sociodemographic factors as covariates. The “Fruchterman-Reingold” algorithm was applied to have data presented in the relative space, among which the variables with stronger relevance remained together, and the variables with weak relevance were mutually exclusive [36]. The pairwise Markov random field model was used to improve the accuracy of the partial correlation network estimated from L1 regularized neighborhood regression. The least absolute contraction and selection operator was used to obtain regularization and control the network sparsity [37]. The Extended Bayesian Information Criterion parameter was adjusted to 0.5 to create a network with greater parsimony and specificity [38]. The network analysis uses Least Absolute Shrinkage and Selection Operator regularized algorithms to obtain the precision matrix (weight matrix). To indicate the importance of each node (variable) in the network, centrality indexes (expected influence and closeness) were also calculated and provided in supplementary materials (Tables S2 and S4). We performed the above analyses using JASP software version 0.14.1 (JASP Team, Amsterdam, The Netherlands).

#### 2.3.2. Multivariate Analysis of Covariance (MANCOVA)

MANCOVA was conducted to examine the associations between combinations of the 24-hour movement guidelines with depression and anxiety. According to participants’ adherence to the 24-hour movement guidelines, they were classified into eight mutually exclusive groups: None, Sleep only, SB only, MVPA only, Sleep + SB, Sleep + MVPA, SB + MVPA, and All three. Pairwise post hoc comparisons (Bonferroni test) were then performed to examine the differences of depression and anxiety levels across these eight groups after adjusting for socio-demographic variables. MANCOVA was performed in SPSS for Windows, version 26.0 (IBM Corp, Armonk, NY, USA). Statistical significance was set at *p* < 0.05 (two-tailed) for interpreting the results.

## 3. Results

### 3.1. Sample Characteristics

The final sample consisted of 1846 participants (mean age = 20.67 ± 1.61, 36.0% males). The proportion of participants meeting the sleep, SB, and MVPA guideline was 69.9%, 68.9%, and 48.5%, respectively. The proportion of combinations of these guidelines varied from 3.6% (MVPA only) to 27.0% (all three). On the PHQ-9 (M = 6.83, SD = 5.19), 63.5% participants reported mild to severe depression symptoms, respectively. On the SAS (M = 41.79, SD = 9.82), 21.8% of participants reported mild to severe anxiety symptoms, respectively. Detailed characteristics are provided in Table 1. 

### 3.2. Individual Associations between Meeting 24-Hour Movement Guidelines with Depression and Anxiety

**Figure 1:**
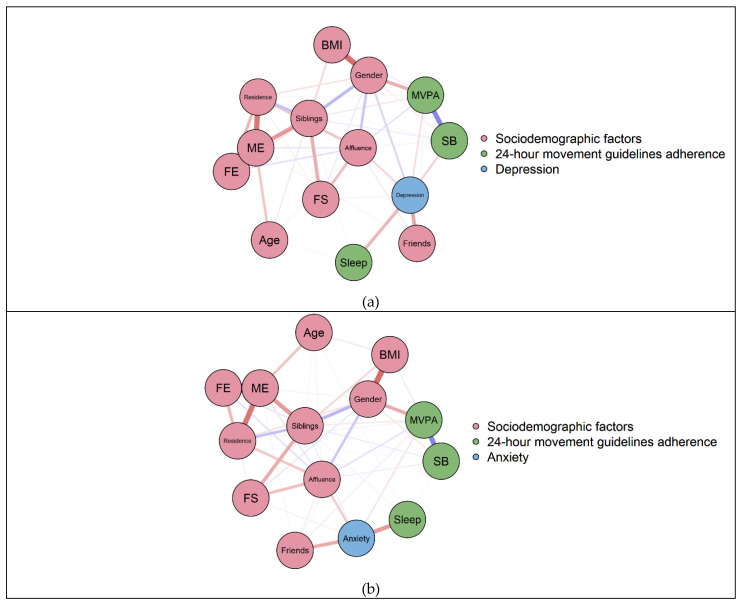
(**a**) Network displaying individual associations between 24-hour movement guidelines and depression. (**b**) Network displaying individual associations between 24-hour movement guidelines and anxiety. Note. Blue edges represent positive associations, and red edges represent negative associations. The thickness of the edges reflects the magnitude of the associations. BMI = body mass index; FS = family structure; FE = father’s education; ME = mother’s education; Siblings = number of siblings; Friends = number of friends; Affluence = perceived family affluence; Sleep = meet the sleep guideline or not; SB = meet the sedentary guideline or not; MVPA = meet the moderate-to-vigorous physical activity guideline or not.

Figure 1 depicts the individual associations between meeting 24-hour movement guidelines with depression and anxiety after adjusting for sociodemographic factors. As shown in Figure 1a, the depression level was inversely correlated with meeting the sleep guideline (−0.15), SB guideline (−0.09), and MVPA guideline (−0.07), respectively. As shown in Figure 1b, the anxiety level was also inversely related to meeting the sleep guideline (−0.21) and the MVPA guideline (−0.05), but not related to meeting the SB guideline (0.00). The weight matrices of the edges of the networks are provided in Supplementary Materials (Tables S1 and S3). 

### 3.3. Combined Associations between Meeting 24-Hour Movement Guidelines with Depression and Anxiety

**Figure 2:**
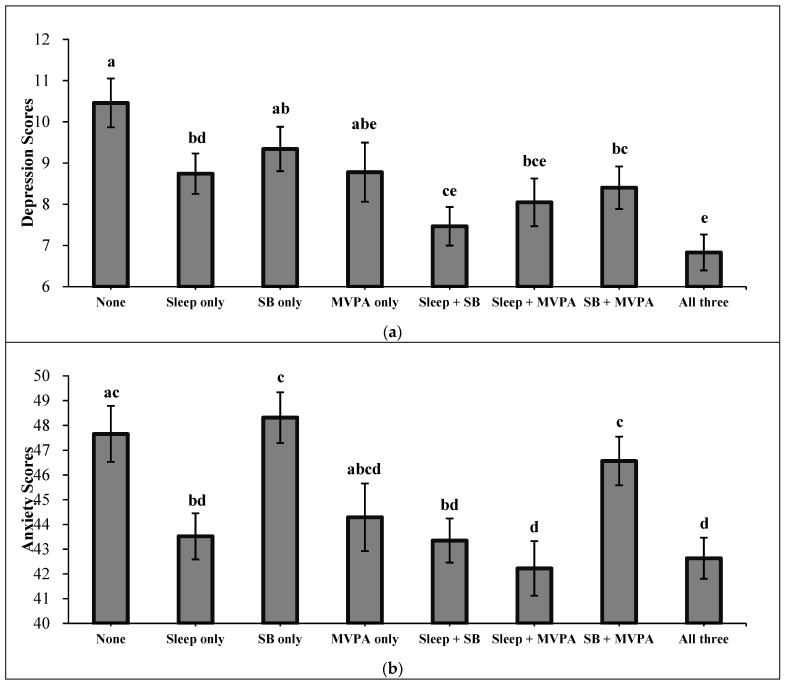
(**a**) Levels of depression according to different combinations of guidelines met. (**b**) Levels of anxiety according to different combinations of guidelines met. Note. Results are from multivariate analysis of covariance and pairwise post-hoc comparisons (Bonferroni test). Letters are denoted to mark the differences between groups. Groups with the same letter are not significantly different and groups that are significantly different get different letters. The error bars represent the 95% confidence intervals of standard error. Results are adjusted for age, gender, body mass index, family structure, parents’ educational level, number of siblings, number of friends, residence, and perceived family affluence. SB = meet the sedentary guideline; MVPA = meet the moderate-to-vigorous physical activity guideline.

Figure 2 shows levels of depression and anxiety across combinations of 24-hour movement guidelines after adjusting for confounding variables. Compared with “None”, depression levels were significantly lower in following combinations: “Sleep only” (*p =* 0.043), “Sleep + SB” (*p* < 0.001), “Sleep + MVPA” (*p* = 0.003), “SB + MVPA” (*p* = 0.008), and “All three” (*p* < 0.001). Generally, as the number of guidelines met increased, the levels of depression showed a downward trend. Compared with “None”, anxiety levels were significantly lower in the following combinations: “Sleep only” (*p* = 0.002), “Sleep + SB” (*p* < 0.001), “Sleep + MVPA” (*p* < 0.001), and “All three” (*p* < 0.001). Detailed results of pairwise post-hoc comparisons are provided in supplementary materials (Tables S5 and S6).

## 4. Discussion

This study investigated the associations between adherence to 24-hour movement guidelines and levels of depression and anxiety by integrating network analysis in the context of the COVID-19 pandemic. We found that meeting the sleep guideline had stronger associations with depression and anxiety than meeting the SB or MVPA guidelines. Compared to meeting none of the guidelines, meeting the sleep guideline only, meeting sleep + SB guidelines, meeting sleep + MVPA guidelines, and meeting all three guidelines were associated with significantly lower levels of depression and anxiety; meeting SB + MVPA guidelines was also associated with a significantly lower level of depression. These findings would deepen our understanding of the role of 24-hour movement behaviors on mental health. Interpretations for the main findings are as follows. 

We identified that sleep guideline adherence was significantly related to lower levels of depression and anxiety. This finding corroborates those of previous literature [39,40]. For instance, a recent study on an adult sample (mean age = 48 ± 14 years) reported that participants meeting the sleep guideline were about twice as likely to have less stress than those who failed to meet [17]. Additionally, Tang et al. found that Chinese college students who slept less than 6 h per night (i.e., not meeting the sleep guideline) reported more depression symptoms than others during the COVID-19 outbreak [8]. Longitudinal data from 29,251 healthy Korean adults also showed that the efficacious sleep duration to reduce future anxiety symptoms was 7–9 h a day [40], which is exactly the recommended sleep duration in the 24-hour movement guidelines [12]. Meeting the sleep guideline means that the sleep duration per day falls within the range considered healthy (e.g., 7–9 h per night recommended for adults aged 18–64 years), while not meeting the sleep guideline includes two possible conditions: insufficient sleep or excessive sleep. A recent study on 28,202 Chinese adults found a U-shaped dose–response relationship between night sleep duration and depression symptoms [41]. Similarly, a meta-analysis of prospective studies reported that too short and long sleep duration were both significantly associated with an increased risk of depression in adults [39]. Inadequate sleep can directly lead to daytime sleepiness and unsuccessful emotion regulation strategies, resulting in more negative and less positive emotions, all of which are risks for psychiatric conditions (e.g., depression and anxiety) [42]. Furthermore, abnormal sleep duration may result from poor sleep quality, while there is robust evidence to support an association between poor sleep quality and depression [43]. All of these may partly account for the associations between the sleep guideline and mental health indicators in the current study. 

Moreover, the sleep guideline yielded a more robust association with depression and anxiety than the SB and MVPA guideline. This finding coincides with a recently published study, which reported that participants meeting the sleep guideline only, any combination of two guidelines, or all three guidelines reported less stress than those meeting none of the guidelines, except for those who met the MVPA or SB guideline only [17]. Our findings also support previous results based on samples of other age groups. For example, a recent longitudinal study on youth concluded that adherence to the sleep guideline, rather than MVPA or SB guideline, was the most consistent predictor of depression symptoms [44]. A systematic review on children and adolescents also suggested that meeting the sleep guideline appeared to be correlated with more mental health benefits than meeting the PA guideline [45]. These findings highlight the relative importance of the sleep guideline in the 24-hour movement guidelines. Meanwhile, considering that sleep occupies a large proportion of time among the three 24-hour movement behaviors, we call for giving priority to the sleep guideline when implementing the 24-hour movement guidelines on campus and encourage those who meet none of the guidelines to start by cultivating a good sleep routine, so as to obtain greater mental health benefits. 

A somewhat surprising finding was that meeting the SB or MVPA guideline alone was not associated with significantly lower levels of depression and anxiety, given the substantial evidence illustrating the desirable impact of limited SB and increased PA on aspects of mental well-being [46,47]. A possible explanation for our results is the use of a self-reported measure of SB and PA, which might have lacked the accuracy needed to measure the duration spent on SB and MVPA. Although meeting the SB or MVPA guidelines showed a relatively weak association with depression and anxiety in this study, meeting SB and MVPA guidelines concurrently was associated with a significantly lower level of depression. Moreover, we found that adherence to more 24-hour movement guidelines was associated with lower risks of depression in a trend of dose-response manner: the depression level decreased as more guidelines were achieved and was the lowest when all three guidelines were met. The same or similar dose-response relationship trend was also reported in previous literature examining the implications of the 24-hour movement guidelines [14,17,48], although their outcomes and population of interest differed from the current study. These findings imply that the benefits of healthy behavior may be superimposed. Therefore, in addition to meeting the sleep guideline, meeting the SB and MVPA guidelines should not be overlooked due to proven multiple health benefits derived from moving more and sitting less [28,49]. Nevertheless, when the outcome was anxiety, the dose–response pattern did not emerge. Owing to the scarcity of studies investigating 24-hour movement guidelines and mental health among adults, the existing evidence is too little to draw a definite conclusion. Further investigations are warranted for a clear explanation of these results.

### 4.1. Strengths and Practical Implications

The novelties of this study include the use of a novel network perspective to understand the associations between adherence to 24-hour movement guidelines and mental health problems among college students. Network analysis presents the innate complexity of these relationships, intuitively, in a graphical format, so we were able to discern unique relationships between 24-hour movement behaviors and mental health problems. More importantly, this study is one of the first to assess the associations in young adults, adding evidence to the implications of the Canadian 24-hour movement guideline in a Chinese adult sample. Considering that the Canadian 24-hour movement guidelines have cultural adaptations to different young populations, this study adds to the evidence on the updates of the guidelines in the future. 

This study also provides some practical implications for protecting mental health in the context of the current pandemic situation. Results of the present study respond to the recent call for adopting healthy movement behaviors to facilitate positive trajectories of mental health following the COVID-19 pandemic [50]. In a global survey during COVID-19-related home confinement, Trabelsi et al. asserted that health education and support for sleep and PA need to be promoted in order to maintain health during the pandemic [7]. In particular, this study underlines the important role of proper sleep duration in coping psychologically with ongoing special events such as this COVID-19 pandemic. However, research indicated that sleep deteriorated in response to the pandemic, and the prevalence of short sleep and long sleep (not meeting the sleep guideline) was higher than that before the pandemic [51,52]. Therefore, it is essential to keep the general population well informed about the importance of sleep and healthy sleep habits through public health education. Based on the existing literature, some recommendations are feasible and effective for individuals to take action and improve sleep [53,54,55]. First, keep in mind that it is normal to perceive lifestyle disruption due to the pandemic, and believe that it is possible to find a balance again. Second, keep a regular sleep–wake schedule and reserve 7–9 h per night for sleep. Third, avoid entertainment or work activities in bed, and reduce screen use before sleep. Fourth, carry out some physical activities, preferably in the daytime. Fifth, try to get natural daylight during the day and have dim light during the evening.

### 4.2. Limitations and Future Directions

Nevertheless, several limitations should be considered in the interpretation of our findings. First, the cross-sectional design precludes confirmation of causality between movement behaviors with depression and anxiety. Physical inactivity, prolonged sitting time, and abnormal sleep duration can also be the consequences of depression or anxiety [56,57], or reciprocal associations exist between these movement behaviors and mental health, as some literature has proposed [58,59]. Second, the self-reported data of movement behaviors and mental health problems, despite PHQ-9 and SAS being psychometrically valid, might have been prone to inaccuracy due to potential recall biases and social desirability. Since accurate assessment of movement behaviors is crucial for defining recommendations for health promotion at a population level, objective measures of movement behaviors, such as pedometers and accelerometers, are preferable in future studies to improve the accuracy of health-related data. Feasible, consumer-grade products (e.g., mobile applications, wearable devices) also make it possible to collect and analyze movement behaviors in a large-scale study [60,61]. Future research is needed to confirm and build upon this study with objective measures of movement behaviors. Third, our study recruited samples using a convenience sampling procedure, so the representativeness of the study sample cannot be guaranteed. Fourth, the Canadian 24-hour movement guidelines also include a quantitative recommendation on the time spent on recreational screen-based SB (≤3 h), which we did not measure in this study. Finally, although we included a series of confounding variables in our study, some other important correlates were not considered, such as dietary behaviors, which are known to be correlated with mental health problems [62]. Accordingly, future research should account for these limitations when drafting a study plan to investigate 24-hour movement guideline adherence and mental health.

## 5. Conclusions

Adherence to the 24-hour movement guidelines was related to lower levels of depression and anxiety among Chinese college students. Greater benefits could be seen when all three guidelines were met. Notably, the benefits were mainly attributed to meeting the sleep guideline. Therefore, promoting adherence to the 24-hour movement guidelines, particularly prioritizing a healthy sleep routine, should be encouraged among college students for better mental health. 

## Figures and Tables

**Table 1:** Sample characteristics.

Variables	Category	*n*	%	Variables	Category/Range	*N*/M	%/SD
Gender	Male	665	36.0	Meeting the MVPA guideline	No	950	51.5
	Female	1181	64.0		Yes	896	48.5
Family structure	Full	1664	90.1	Combinations of guidelines met	None	119	6.4
	Divorced	117	6.3		Sleep only	260	14.1
	Other	65	3.5		SB only	168	9.1
Father’s education	Middle school or below	891	48.3		MVPA only	67	3.6
	High school	636	34.5		Sleep + SB	403	21.8
	College or university	254	13.8		Sleep + MVPA	129	7.0
	Master or above	65	3.5		Sleep + MVPA	201	10.9
Mother’s education	Middle school or below	1086	58.8		Sleep + SB + MVPA	499	27.0
	High school	560	30.3	Depression symptoms	Minimal	673	36.5
	College or university	158	8.6		Mild	738	40.0
	Master or above	42	2.3		Moderate	279	15.1
Number of siblings	None	639	34.6		Moderately severe	108	5.9
	One or more	1207	65.4		Severe	48	2.6
Number of friends	None	29	1.6	Anxiety symptoms	Minimal	1462	79.2
	One to two	608	32.9		Mild	277	15.0
	Three to five	964	52.2		Moderate	89	4.8
	Six or more	245	13.3		Severe	18	1.0
Residence	Urban	1278	69.2	**Variables**	**Range**	**M**	**SD**
	Rural	568	30.8	Age (years)	18–26	20.67	1.61
Meeting the sleep guideline	No	555	30.1	BMI (kg/m^2^)	10–44	20.27	2.88
	Yes	1291	69.9	Perceived family affluence	1–10	5.71	1.64
Meeting the SB guideline	No	575	31.1	Depression symptoms	0–27	6.83	5.19
	Yes	1271	68.9	Anxiety symptoms	25–85	41.79	9.82

## Data Availability

The data analyzed during the current study are available from the corresponding author on reasonable request.

## Supplementary Materials

The following are available online at https://www.mdpi.com/article/10.3390/healthcare9091166/s1, Table S1: Weight matrix of the network for depression, Table S2: Centrality measures per variable in the network for depression, Table S3: Weight matrix of the network for anxiety, Table S4: Centrality measures per variable in the network for anxiety, Table S5: Results of pairwise post-hoc comparisons (Depression), Table S6: Results of pairwise post-hoc comparisons (Anxiety).
