# Supplementary material for: Sleep as a Priority: 24-Hour Movement Guidelines and Mental Health of Chinese College Students during the COVID-19 Pandemic
Source: Healthcare (Basel). 2021 Sep 6;9(9):1166. doi: 10.3390/healthcare9091166 (PMC8468601; doi:10.3390/healthcare9091166)
Supplement: Supplementary file 1 [file healthcare-09-01166-s001.zip › healthcare-1347034-supplementary.pdf]

Supplementary Material

Table S1. Weight matrix of the network for depression

Variable	Gender	Age	Affluence	Friends	Siblings	Residence	FS	FE	ME	BMI	*Sleep*	*SB*	*MVPA*	Depression
Gender	0.00	0.00	0.12	0.00	0.15	−0.06	0.04	0.00	0.02	−0.31	0.00	−0.04	−0.17	0.08
Age	0.00	0.00	−0.02	0.00	−0.01	0.00	0.00	0.00	−0.12	0.04	−0.01	0.00	0.00	0.00
Affluence	0.12	−0.02	0.00	0.04	0.00	−0.12	−0.13	0.06	0.05	0.00	0.00	0.02	0.06	−0.08
Friends	0.00	0.00	0.04	0.00	0.00	0.00	0.00	0.00	0.02	0.00	0.00	0.00	0.02	−0.19
Siblings	0.15	−0.01	0.00	0.00	0.00	0.13	−0.17	−0.06	−0.21	−0.08	0.02	0.04	−0.05	0.02
Residence	−0.06	0.00	−0.12	0.00	0.13	0.00	−0.03	−0.15	−0.29	0.00	0.00	0.01	−0.02	0.00
FS	0.04	0.00	−0.13	0.00	−0.17	−0.03	0.00	0.00	0.00	0.00	0.00	0.00	0.00	0.02
FE	0.00	0.00	0.06	0.00	−0.06	−0.15	0.00	0.00	0.54	0.00	0.00	0.00	0.00	0.00
ME	0.02	−0.12	0.05	0.02	−0.21	−0.29	0.00	0.54	0.00	0.00	0.00	0.00	0.00	0.00
BMI	−0.31	0.04	0.00	0.00	−0.08	0.00	0.00	0.00	0.00	0.00	0.00	0.00	0.04	0.00
Sleep	0.00	−0.01	0.00	0.00	0.02	0.00	0.00	0.00	0.00	0.00	0.00	0.00	0.00	−0.15
SB	−0.04	0.00	0.02	0.00	0.04	0.01	0.00	0.00	0.00	0.00	0.00	0.00	0.25	−0.09
MVPA	−0.17	0.00	0.06	0.02	−0.05	−0.02	0.00	0.00	0.00	0.04	0.00	0.25	0.00	−0.07
Depression	0.08	0.00	−0.08	−0.19	0.02	0.00	0.02	0.00	0.00	0.00	−0.15	−0.09	−0.07	0.00

Note. Main variables are in italic.

Table S2. Centrality measures per variable in the network for depression

Variable	Expected influence	Closeness
Gender	−0.18	1.81
Age	0.48	0.91
Affluence	0.06	−1.36
Friends	−0.44	1.31
Siblings	−0.63	−0.03
Residence	−0.77	0.40
FS	−0.02	−1.46
FE	−1.65	0.15
ME	2.10	−0.09
BMI	0.51	0.52
Sleep	−0.12	−1.56
SB	1.27	−0.46
MVPA	0.78	0.07
Depression	−1.38	−0.22

Table S3. Weight matrix of the network for anxiety

Variable	Gender	Age	Affluence	Friends	Siblings	Residence	FS	FE	ME	BMI	*Sleep*	*SB*	*MVPA*	Anxiety
Gender	0.00	0.00	0.12	−0.02	0.15	−0.06	0.05	0.00	0.03	−0.31	0.00	−0.05	−0.18	0.00
Age	0.00	0.00	−0.02	0.00	−0.02	0.00	0.00	0.00	−0.12	0.04	−0.02	0.00	0.00	0.00
Affluence	0.12	−0.02	0.00	0.04	0.00	−0.12	−0.14	0.06	0.05	0.00	0.00	0.03	0.06	−0.11
Friends	−0.02	0.00	0.04	0.00	0.00	0.00	0.00	0.00	0.02	0.00	0.00	0.00	0.03	−0.18
Siblings	0.15	−0.02	0.00	0.00	0.00	0.13	−0.18	−0.06	−0.21	−0.08	0.02	0.04	−0.05	0.04
Residence	−0.06	0.00	−0.12	0.00	0.13	0.00	−0.03	−0.15	−0.29	0.00	0.00	0.01	−0.03	0.00
FS	0.05	0.00	−0.14	0.00	−0.18	−0.03	0.00	0.00	0.00	0.00	0.00	0.00	0.00	0.03
FE	0.00	0.00	0.06	0.00	−0.06	−0.15	0.00	0.00	0.54	0.00	0.00	0.00	0.00	0.00
ME	0.03	−0.12	0.05	0.02	−0.21	−0.29	0.00	0.54	0.00	0.00	0.00	−0.01	0.00	0.00
BMI	−0.31	0.04	0.00	0.00	−0.08	0.00	0.00	0.00	0.00	0.00	0.00	0.00	0.04	0.00
Sleep	0.00	−0.02	0.00	0.00	0.02	0.00	0.00	0.00	0.00	0.00	0.00	0.01	0.00	−0.21
SB	−0.05	0.00	0.03	0.00	0.04	0.01	0.00	0.00	−0.01	0.00	0.01	0.00	0.26	0.00
MVPA	−0.18	0.00	0.06	0.03	−0.05	−0.03	0.00	0.00	0.00	0.04	0.00	0.26	0.00	−0.05
Anxiety	0.00	0.00	−0.11	−0.18	0.04	0.00	0.03	0.00	0.00	0.00	−0.21	0.00	−0.05	0.00

Note. Main variables are in italic.

Table S4. Centrality measures per variable in the network for anxiety

Variable	Expected influence	Closeness
Gender	−0.58	1.26
Age	−0.04	−1.26
Affluence	0.38	1.32
Friends	0.08	−1.58
Siblings	−0.33	1.10
Residence	−1.52	0.52
FS	−0.55	0.36
FE	1.95	0.21
ME	0.48	0.89
BMI	−0.70	0.05
Sleep	−0.26	−1.42
SB	1.64	−0.94
MVPA	0.82	−0.01
Anxiety	−1.37	−0.50

Table S5. Results of pairwise post-hoc comparisons (Depression)

**Groups (I)**	**Groups (J)**	**Mean difference (I-J)**	**95%CI**		*p*
			Lower limit	Upper limit	
None	Sleep only	1.72	0.02	3.41	**0.043**
	SB only	1.12	−0.72	2.95	1.000
	MVPA only	1.68	−0.67	4.02	0.707
	Sleep + SB	2.99	1.39	4.59	**0.000**
	Sleep + MVPA	2.41	0.47	4.35	**0.003**
	SB + MVPA	2.06	0.28	3.83	**0.008**
	All three	3.63	2.05	5.20	**0.000**
Sleep only	SB only	−0.60	−2.12	0.92	1.000
	MVPA only	−0.04	−2.15	2.07	1.000
	Sleep + SB	1.28	0.06	2.49	**0.030**
	Sleep + MVPA	0.70	−0.95	2.34	1.000
	SB + MVPA	0.34	−1.10	1.78	1.000
	All three	1.91	0.73	3.10	**0.000**
SB only	MVPA only	0.56	−1.66	2.78	1.000
	Sleep + SB	1.88	0.47	3.29	**0.001**
	Sleep + MVPA	1.30	−0.50	3.09	0.668
	SB + MVPA	0.94	−0.67	2.55	1.000
	All three	2.51	1.13	3.89	**0.000**
MVPA only	Sleep + SB	1.31	−0.72	3.35	1.000
	Sleep + MVPA	0.73	−1.58	3.05	1.000
	SB + MVPA	0.38	−1.79	2.55	1.000
	All three	1.95	−0.05	3.95	0.066
Sleep + SB	Sleep + MVPA	−0.58	−2.13	0.96	1.000
	SB + MVPA	−0.94	−2.26	0.39	0.769
	All three	0.64	−0.41	1.68	1.000
Sleep + MVPA	SB + MVPA	−0.35	−2.08	1.37	1.000
	All three	1.22	−0.30	2.73	0.340
SB + MVPA	All three	1.57	0.29	2.85	**0.004**

Note: The significant *p* values are bold.

Table S6. Results of pairwise post-hoc comparisons (Anxiety)

**Groups (I)**	**Groups (J)**	Mean difference **(I-J)**	95%CI		*p*
			Lower limit	Upper limit	
None	Sleep only	4.14	0.92	7.36	**0.002**
	SB only	−0.66	−4.15	2.84	1.000
	MVPA only	3.37	−1.10	7.83	0.514
	Sleep + SB	4.31	1.27	7.35	**0.000**
	Sleep + MVPA	5.43	1.74	9.13	**0.000**
	SB + MVPA	1.09	−2.29	4.47	1.000
	All three	5.03	2.02	8.03	**0.000**
Sleep only	SB only	−4.80	−7.68	−1.91	**0.000**
	MVPA only	−0.77	−4.79	3.25	1.000
	Sleep + SB	0.17	−2.15	2.49	1.000
	Sleep + MVPA	1.30	−1.83	4.42	1.000
	SB + MVPA	−3.05	−5.80	−0.30	**0.015**
	All three	0.89	−1.37	3.14	1.000
SB only	MVPA only	4.02	−0.20	8.25	0.083
	Sleep + SB	4.97	2.29	7.65	**0.000**
	Sleep + MVPA	6.09	2.68	9.50	**0.000**
	SB + MVPA	1.75	−1.32	4.81	1.000
	All three	5.68	3.06	8.31	**0.000**
MVPA only	Sleep + SB	0.94	−2.93	4.82	1.000
	Sleep + MVPA	2.07	−2.34	6.47	1.000
	SB + MVPA	−2.28	−6.40	1.85	1.000
	All three	1.66	−2.15	5.47	1.000
Sleep + SB	Sleep + MVPA	1.12	−1.82	4.07	1.000
	SB + MVPA	−3.22	−5.75	−0.70	**0.002**
	All three	0.71	−1.28	2.71	1.000
Sleep + MVPA	SB + MVPA	−4.34	−7.63	−1.06	**0.001**
	All three	−0.41	−3.29	2.48	1.000
SB + MVPA	All three	3.94	1.49	6.38	**0.000**

Note: The significant *p* values are bold.
